# Supplementary material for: Lacticaseibacillus paracasei GM-080 Ameliorates Allergic Airway Inflammation in Children with Allergic Rhinitis: From an Animal Model to a Double-Blind, Randomized, Placebo-Controlled Trial
Source: Cells. 2023 Feb 28;12(5):768. doi: 10.3390/cells12050768 (PMC10000597; doi:10.3390/cells12050768)
Supplement: Supplementary file 1 [file cells-12-00768-s001.zip › cells-2195174-supplementary.pdf]

Lacticaseibacillus paracasei GM-080 Ameliorates Allergic Airway Inflammation in Children with Allergic Rhinitis: From an Animal Model to a Double-Blind, Randomized, Placebo-Controlled Trial

En-Kwang Lin, Wen-Wei Chang, Jhih-Hua Jhong, Wan-Hua Tsai, Chia-Hsuan Chou and I-Jen Wang

Supporting information

Table S1 CRISPR loci, prophage-like clusters, and bacteriocin gene in the GM-080 genome.

CRISPR loci

Position	Length	Repeat consensus	Repeat length	Spacers number
1076933-1078354	1421	GCTCTTGAACTGATTGATTCGACATCTACCTGAGAC	36	21

Prophage

Locate	Length	Completeness[a]	Score	Region Position	Protein Number	GC (%)
Chr	44.6Kb	intact	130	760-45436 info_outline	63	45.05
	30.7Kb	intact	130	798774-829567 info_outline	28	43.58
	13.6Kb	incomplete	20	1781477-1795130 info_outline	12	45.38
	31.5Kb	intact	130	2386341-2417905 info_outline	43	44.26
	34.1Kb	intact	141	2757738-2791893 info_outline	47	45.64
	39.6Kb	intact	144	2867742-2907382 info_outline	54	45.29
	64.6Kb	intact	137	3075976-3140658 info_outline	95	44.55

Bacteriocin

Locate	AOI[b]	start	end	Class	Organism	Uniprot	PMID	Sequence
Chr	AOI_01	1262514	1291655	143.2;LSEI_2386	Carnobacterium piscicola	P38582.1	-	MDIKSQTLYLNLSEAYKDPEVKANEFLSKLVVQCAGKLTASNSENSYIEVISLLSRGISSYYLSHKRIIPSSMLTIYTQIQKDIKNGNIDTEKLRKYEIAKGLMSVPYIY

[a] Prediction of prophage region: intact (score > 90), questionable (score 70–90) or incomplete (score < 70)

[b] AOI, area of interest. These are potential clusters responsible for the production of RiPPs and other bacteriocins.

Table S2 Minimum inhibitory concentrations (MICs) of *L. paracasei* GM-080 toward eight antimicrobials and their microbiological cutoffs.

Antbiotics	EFSA cut-off [a, b]	GM-080 MIC[a]
Ampicillin	4	2~4
Gentamicin	32	2~4
Kanamycin	64	32~64
Streptomycin	64	8~16
Erythromycin	1	0.25
Clindamycin	1	0.0625~0.125
Tetracycline	4	1
Chloramphenicol	4	4

[a] g/L

[b] according to ref 17

Table S3 EggNOG functional annotations of *L. paracasei* GM-080.

Functional Categories	Gene	egg NOG annotation	Brite		
Amino acid transport and metabolism (E)	dppA	D-aminopeptidase	Bacterial chemotaxis		
	pepA	M42 glutamyl aminopeptidase	Glutathione metabolism		
	yxeO	ABC transporter	L-cystine transport		
Carbohydrate transport and metabolism (G)	agaD	PTS system mannose/fructose/sorbose family IID component	Galactose metabolism		
	kduI	Catalyzes the isomerization of 5-dehydro-4-deoxy-D- glucuronate to 3-deoxy-D-glycero-2,5-hexodiulosonate	Pentose and glucuronate interconversions		
	kdgK	pfkB family carbohydrate kinase	Pentose phosphate pathway		
	ahaA	PTS system fructose IIA component	Biosynthesis of secondary metabolites	Fructose and mannose metabolism	Starch and sucrose metabolism
	xylP	MFS/sugar transport protein	Glycosidases		
	lacE	Phosphotransferase system, EIIC	Galactose metabolism		
	lacG	Belongs to the glycosyl hydrolase 1 family		lactose/L-arabinose transport	
	lacF	PTS system, Lactose/Cellobiose specific IIA subunit			
Replication, recombination and repair (L)	recT	RecT family	DSBR (double strand breaks repair)		
	pi112	PDDEXK-like domain of unknown function (DUF3799)	prophage pi1 protein 12		
	tnpA1	PFAM transposase, IS204 IS1001 IS1096 IS1165 family protein	Chromosome and associated proteins		
	tnp1216	DDE domain			
	cas2	CRISPR	CRISPR-associated protein Cas2	Universal Cas proteins	
	cas1	CRISPR	CRISPR-associated protein Cas1		
	cas9	CRISPR	CRISPR-associated endonuclease Csn1		
	int3	Belongs to the 'phage' integrase family	Spliceosome		
	pi346	IstB-like ATP binding protein	DNA replication protein DnaC		
	tnpR	Resolvase, N terminal domain			
	is18	Integrase core domain	putative transposase		
	yqaJ	YqaJ-like viral recombinase domain			
	rusA	Endodeoxyribonuclease RusA	DNA repair and recombination proteins		
Cell wall/membrane/envelope biogenesis (M)	rfbP	Bacterial sugar transferase	undecaprenyl-phosphate galactose phosphotransferase	Lipopolysaccharide biosynthesis proteins	
	rgpAc	Domain of unknown function (DUF1972)	rgpA; rhamnosyltransferase	Lipopolysaccharide biosynthesis proteins	
	mprF	lysyltransferase activity	Two-component system		

Table S4. Putative IMa and CpG-containing ODN[b] contents in *L. paracasei* strains.

			GM-080	BCRC 16100	L9[c]	HD1.7[c]	Lpc10[c]
Code	Sequence	Genome size (bp)	3,140,923	3,029,023	3,076,440	3,039,280	3,052,120
IM1	TTAGGG	Frequency	292	276	187	184	193
		No. copies per 10^6 bases	93	91.1	60.8	60.5	63.2
IM2	TTTCGTTT	Frequency	103	78	54	52	59
		No. copies per 10^6 bases	32.8	25.8	17.6	17.1	19.3
IM3	TCAAGCTTGA	Frequency	4	4	3	2	3
		No. copies per 10^6 bases	1.3	1.3	1.0	0.7	1.0
ODN2216	GACGATCGTC	Frequency	1	3	ND[d]	ND	ND
		No. copies per 10^6 bases	0.3	1	ND	ND	ND
ODN2336	ACGACGTCGT	Frequency	1	1	ND	ND	ND
		No. copies per 10^6 bases	0.3	0.3	ND	ND	ND

[a] ODN, oligonucleotides.

[b] IM, immunosuppressive motif

[c] Information of three reference *Lactobacillus* strains were obtained from Mazhary Z et al., Inform. Med. Unlocked. 2020;18:100280.

[d] ND, not determined
